# Supplementary material for: Structures of active Hantaan virus polymerase uncover the mechanisms of Hantaviridae genome replication
Source: Nat Commun. 2023 May 23;14:2954. doi: 10.1038/s41467-023-38555-w (PMC10206067; doi:10.1038/s41467-023-38555-w)
Supplement: Supplementary file 1 — Supplementary information [file 41467_2023_38555_MOESM1_ESM.pdf]

SUPPLEMENTARY INFORMATION

Structures of active Hantaan virus polymerase uncover the mechanisms of *Hantaviridae* genome replication

Quentin Durieux Trouilleton et *al.*

Supplementary Figure 1

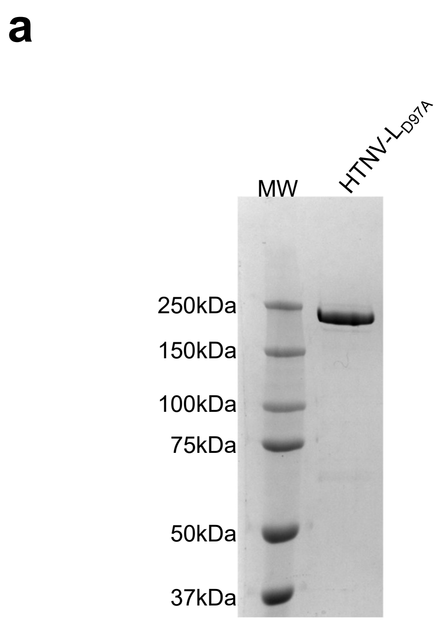

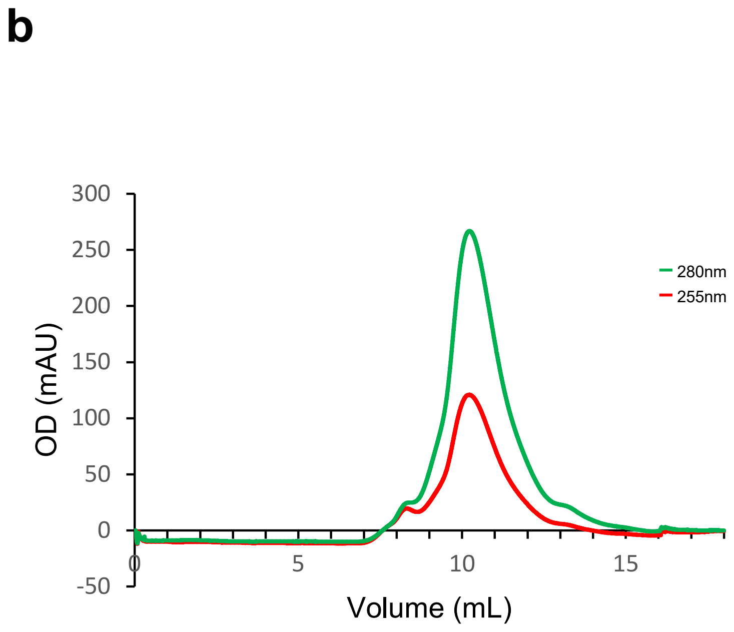

Supplementary Figure 1 HTNV-L_{D97A} purification

a 6% SDS-PAGE gel of HTNV-L_{D97A} after gel filtration. The molecular weight (MW) marker is indicated. **b** Elution profile of gel filtration. Absorbances at 280nm and 255nm are displayed and respectively colored in green and red.

Supplementary Figure 2

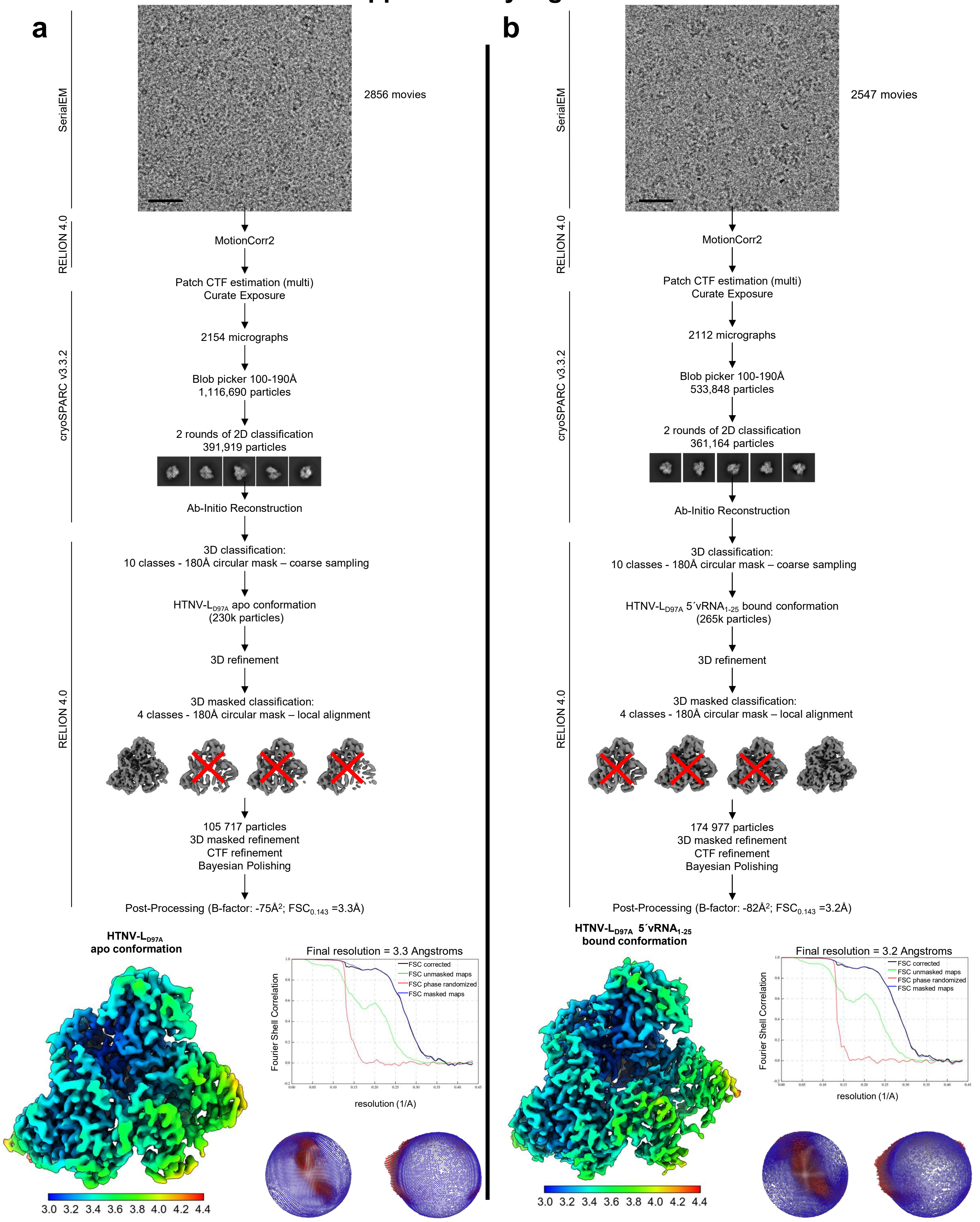

Supplementary Figure 2 Image processing strategy to obtain apo and 5′vRNA$_{1-25}$-bound cryo-EM maps

Schematic representation of image processing used to obtain the apo (**a**) and the 5′vRNA$_{1-25}$-bound (**b**) HTNV-L$_{D97A}$ cryo-EM maps. For both image processing, a representative image is displayed. The scale bar corresponds to 400 nm. 2D class averages, 3D class averages and the final reconstruction are displayed. Electron density maps are colored according to the local resolution. Fourier Shell Correlation curves (FSC) and angular distribution of particles used in the final reconstruction are displayed.

Supplementary Figure 3

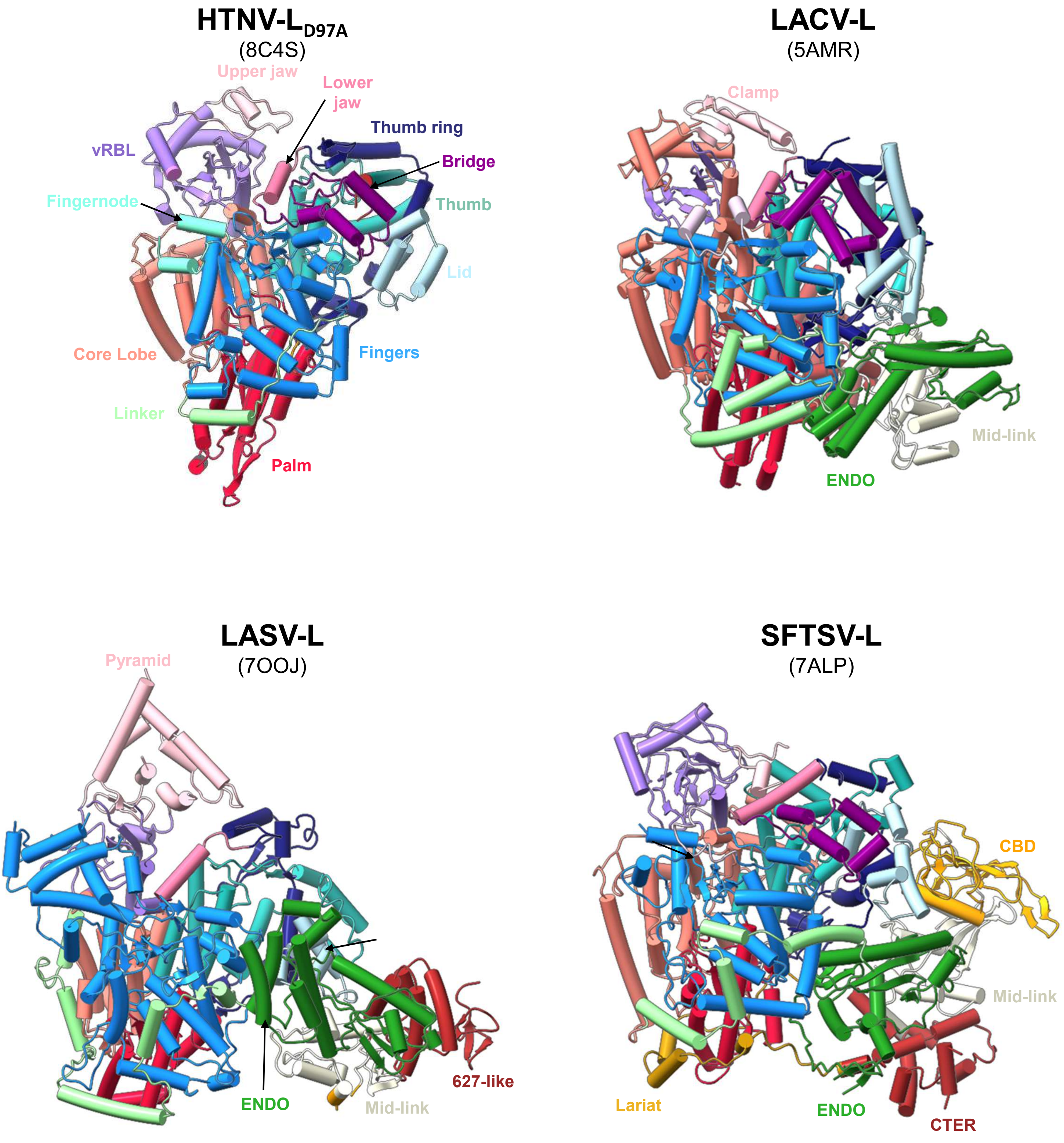

Supplementary Figure 3: Comparison of *Bunyavirales* polymerases.

Polymerase structures of apo Hantaan virus (HTNV-L), La Crosse virus (LACV-L), Severe Fever with Thrombocytopenia Syndrome virus (SFTSV-L) and Lassa virus (LASV-L). Domains are colored as in **Fig. 1** for all polymerase cores. The endonuclease (ENDO), the mid-link, the cap-binding domain (CBD) and the C-terminal domains are respectively colored in green, beige, orange and brown.

Supplementary Figure 4

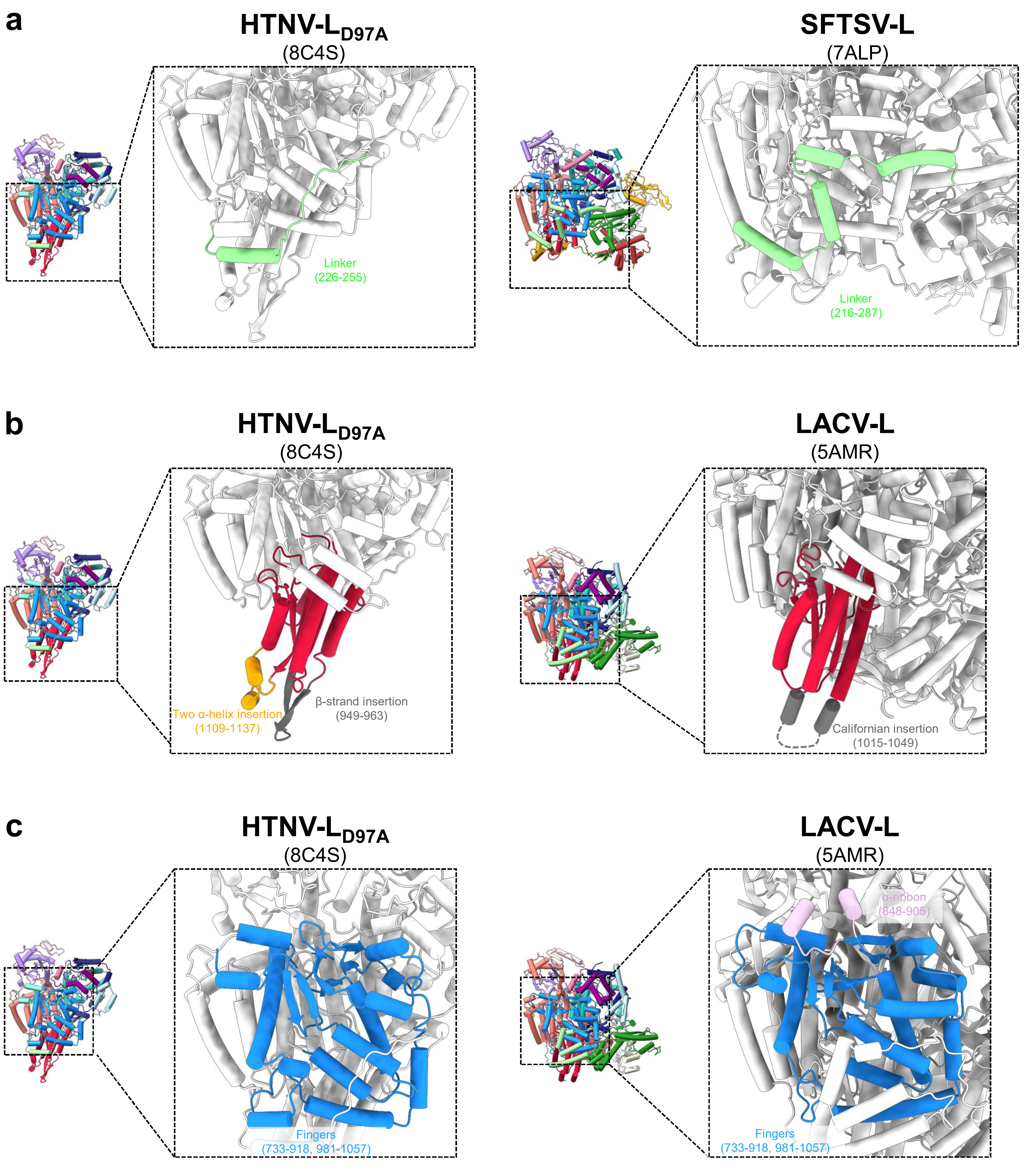

Supplementary Figure 4: Insertions and deletions in HTNV-L$_{D97A}$ compared to other *Bunyavirales* polymerases

a zoom on the linker region of Hantaan virus polymerase (HTNV-L$_{D97A}$) compared to Severe Fever with Thrombocytopenia Syndrome virus polymerase (SFTSV-L). The proteins are shown side-by-side as white cartoon with the linker region in green.

b zoom on the palm domain insertions of HTNV-L$_{D97A}$ compared to La Crosse virus polymerase (LACV-L). Both proteins are shown as white cartoon except the palm that is shown in red and the insertions that are colored differently. HTNV-L$_{D97A}$ two α-helical insertion that is specific to hantaviruses is shown in orange. HTNV-L$_{D97A}$ two β-strand insertion and its corresponding LACV-L α-helical Californian insertion are shown in grey.

c zoom on the finger domain of HTNV-L$_{D97A}$ and LACV-L colored in blue. HTNV-L$_{D97A}$ lacks the α-ribbon insertion that is present in other *Bunyavirales* polymerases and shown on LACV-L in light pink.

Supplementary Figure 5

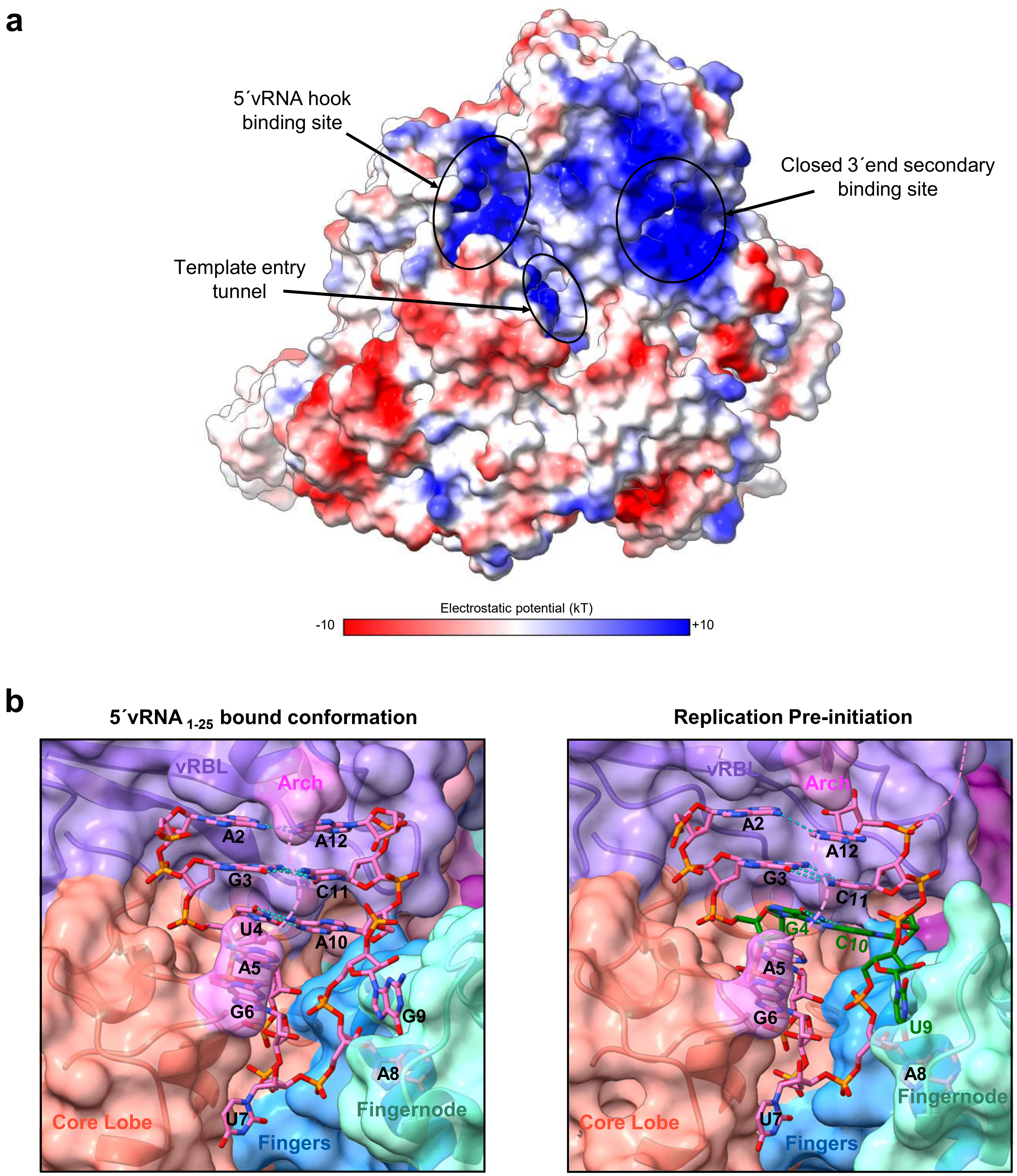

Supplementary Figure 5: 5'vRNA binding site

a Electrostatic potential of HTNV-L_{D97A} apo showing the 5'vRNA hook binding site and the location of the 3' secondary binding site that is closed in the apo conformation.

b comparison of wild type 5'vRNA (5'$vRNA_{1-25}$) and mutated 5'**RNA** (5'mut) binding. The 5'$vRNA_{1-25}$ and the 5'mut are represented in pink and the hydrogen bonds linking the bases of the hook are indicated. Mutated nucleotides are shown in dark green. HTNV-L_{D97A} is shown as a transparent surface to visualize the binding cavity. Regions involved in the binding site formation are labeled and shown as cartoon. Parts of the arch that is disordered is indicated with a dotted line.

Supplementary Figure 6

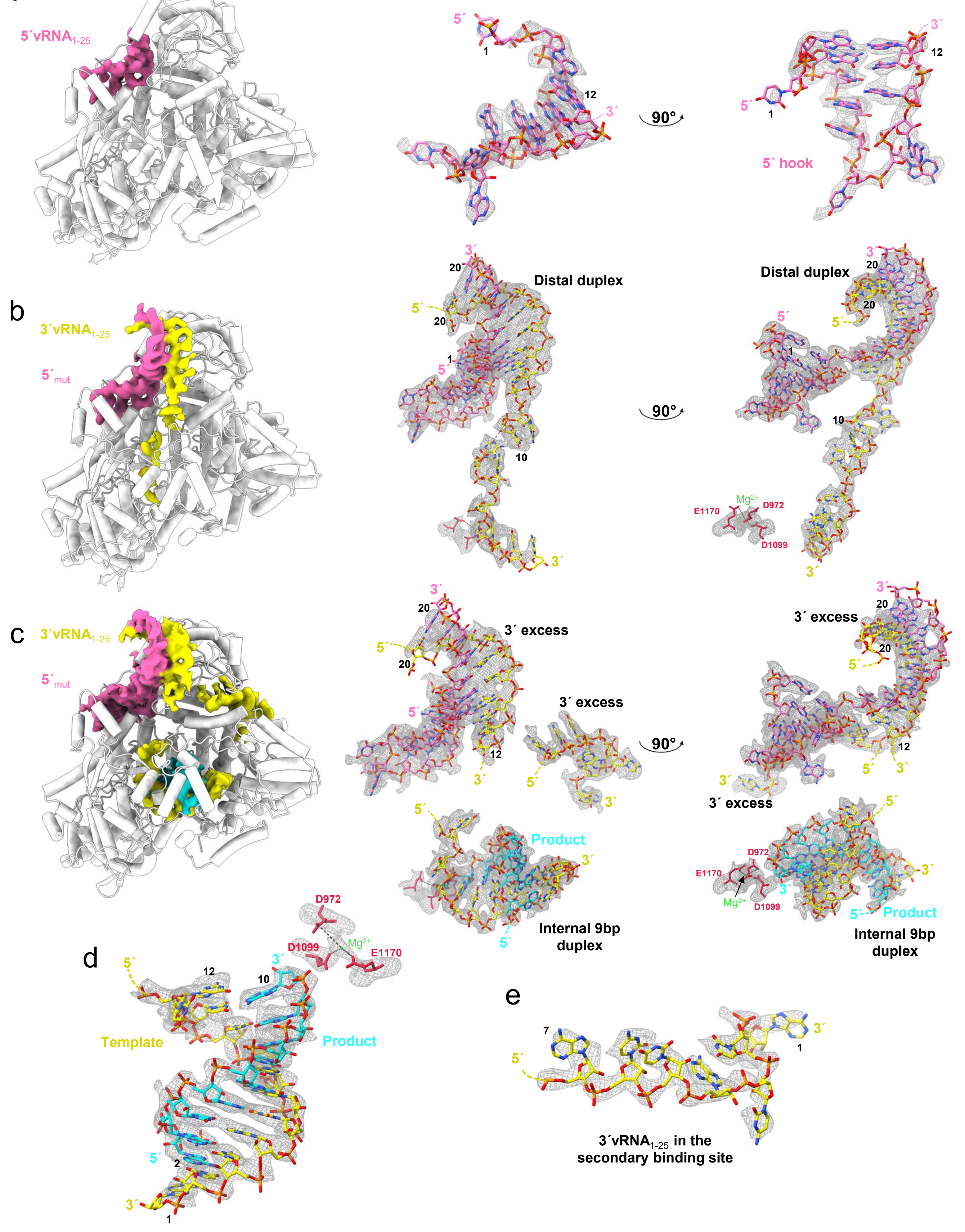

Supplementary Figure 6 RNA densities

a, b, c Densities corresponding to the RNA of the 5'vRNA$_{1-25}$-bound HTNV-L$_{D97A}$ model (**a**), the pre-initiation model (**b**) and the elongation model (**c**). On the left, global view of the models shown as white cartoon with electron densities corresponding to the RNA shown as colored surface. The 5'vRNA$_{1-25}$ or 5'mut are shown in pink, the 3'vRNA$_{1-25}$ in yellow and the product in cyan. On the right, zoom on the RNA models shown as sticks and the electron density displayed as mesh. Two perpendicular views are shown. Nucleotide numbering is indicated. The residues of the active site that coordinate the magnesium ion are also shown and their electron density is displayed as mesh. **d** Focus on the template/product in the active site cavity in HTNV-L$_{D97A}$ replication elongation map. The nucleotides are shown as stick and the electron density is shown as mesh. Nucleotide numbering is indicated. The residues of the active site that coordinate the magnesium ion and the magnesium ion itself are displayed and their corresponding electron density is shown as mesh. **e** Focus on the 3'vRNA secondary binding site in HTNV-L$_{D97A}$ replication elongation map. The nucleotides are shown as stick and the electron density is shown as mesh. Nucleotide numbering is indicated.

Supplementary Figure 7

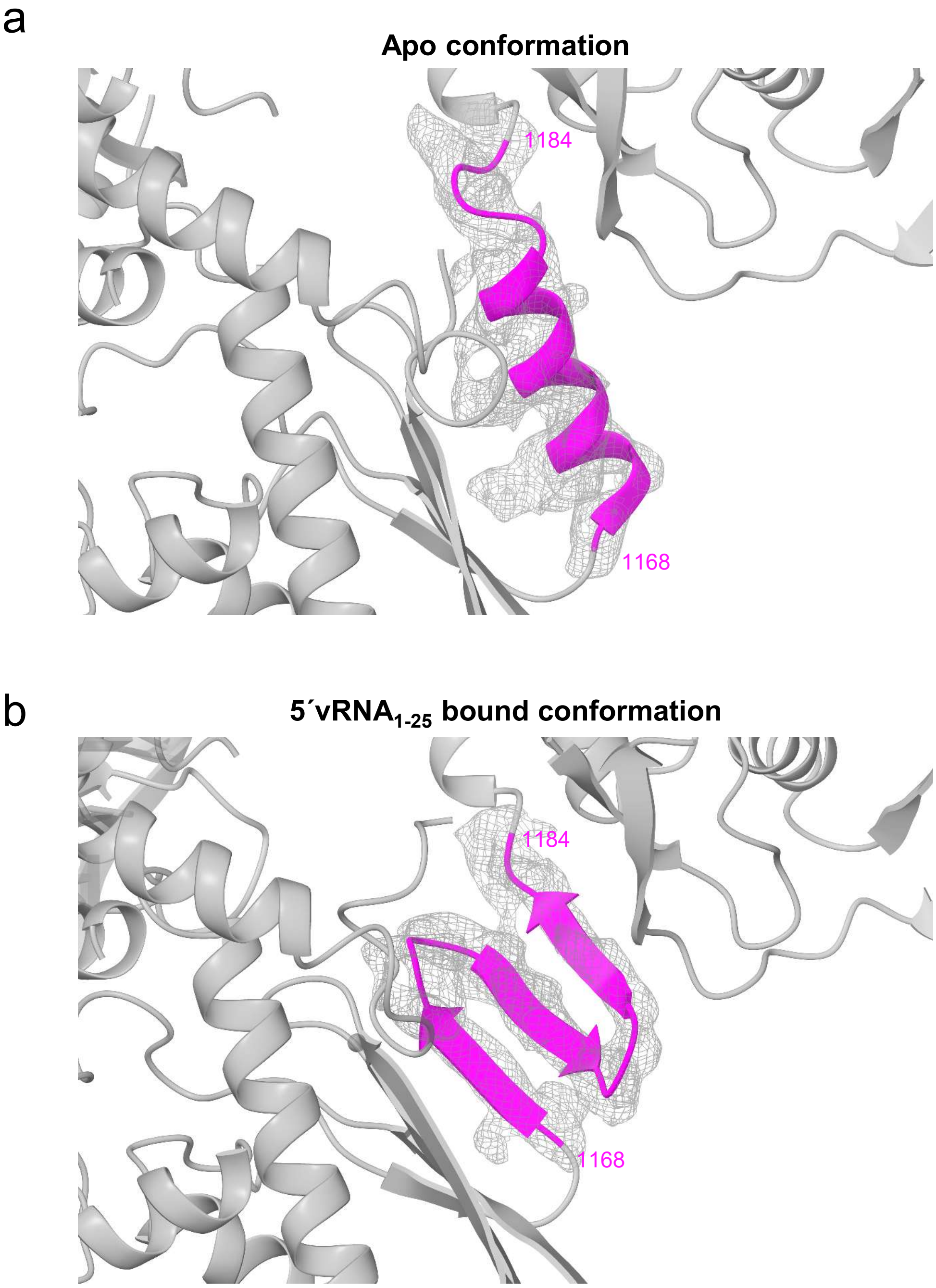

Supplementary figure 7 Electron density corresponding to motif E

The electron density that corresponds to motif E is displayed as mesh for the apo map (**a**) and the 5′vRNA-bound map (**b**). The model is shown in white except for motif E that is shown in magenta.

.

Supplementary Figure 8

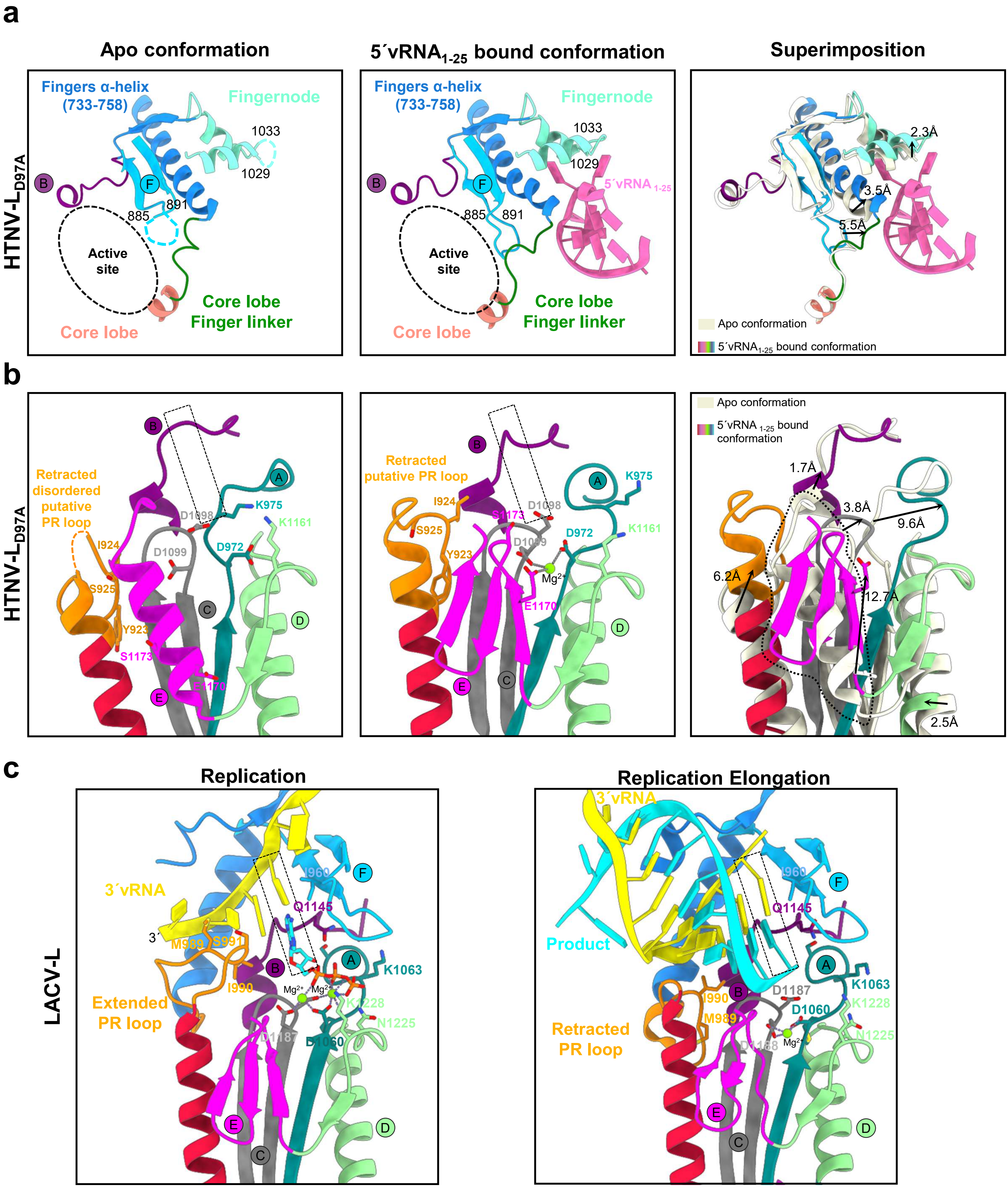

Supplementary Figure 8 Conformational change in HTNV-L$_{D97A}$ active site triggered by 5'vRNA$_{1-25}$ binding and comparison with La Crosse virus polymerase (LACV-L) active site

a Zoom on the 5'vRNA binding site. The fingernode, the finger helix 733-758 and the core lobe finger linker that directly bind to the 5'vRNA hook are displayed as cartoon and respectively colored in cyan, blue and green. Motif F and B that reorganize upon 5'vRNA$_{1-25}$ binding are respectively shown as light blue and purple. Motif F is partially disordered in the apo state as shown by a dotted line. The active site position is indicated as a dotted line. Right panel: overlay of the apo model (in grey) and the 5'vRNA$_{1-25}$-bound model (colored as on the middle panel). Direction of movements are indicated with arrows. Distances between the apo and the 5'vRNA$_{1-25}$-bound maps are indicated in specific places to quantify the movements. **b** Zoom on the active site of HTNV-L$_{D97A}$ in the apo and the 5'vRNA$_{1-25}$-bound map. The motifs and the putative prime-and-realign (PR) loop are shown as cartoon, colored as in **Fig. 2** and labeled. Important residues of the motifs are shown as sticks for both maps, allowing for comparison of residue positions in both maps. The magnesium ion that is bound in the 5'vRNA$_{1-25}$-bound map is shown as a green dot and its coordination is indicated with dotted lines. Right panel: overlay of the apo model (in grey) and the 5'vRNA$_{1-25}$-bound model (colored as on the middle panel). Distances between the apo and the 5'vRNA$_{1-25}$-bound maps are indicated in specific places to quantify the movements. **c** Zoom on the active site of LACV-L at initiation and elongation, enabling the comparison with the active site of HTNV-L$_{D97A}$ in **b**. The motifs and the putative prime-and-realign (PR) loop are shown as cartoon and colored as in **b**. Motif residues that are equivalent to the ones shown in HTNV-L$_{D97A}$ in **b** are shown as sticks. The template and product RNA are respectively shown as yellow and blue. The incoming ATP in position +1 of the active site at initiation is displayed. Magnesium ions are shown as green sticks and their coordination shown as dotted lines.

Supplementary Figure 9

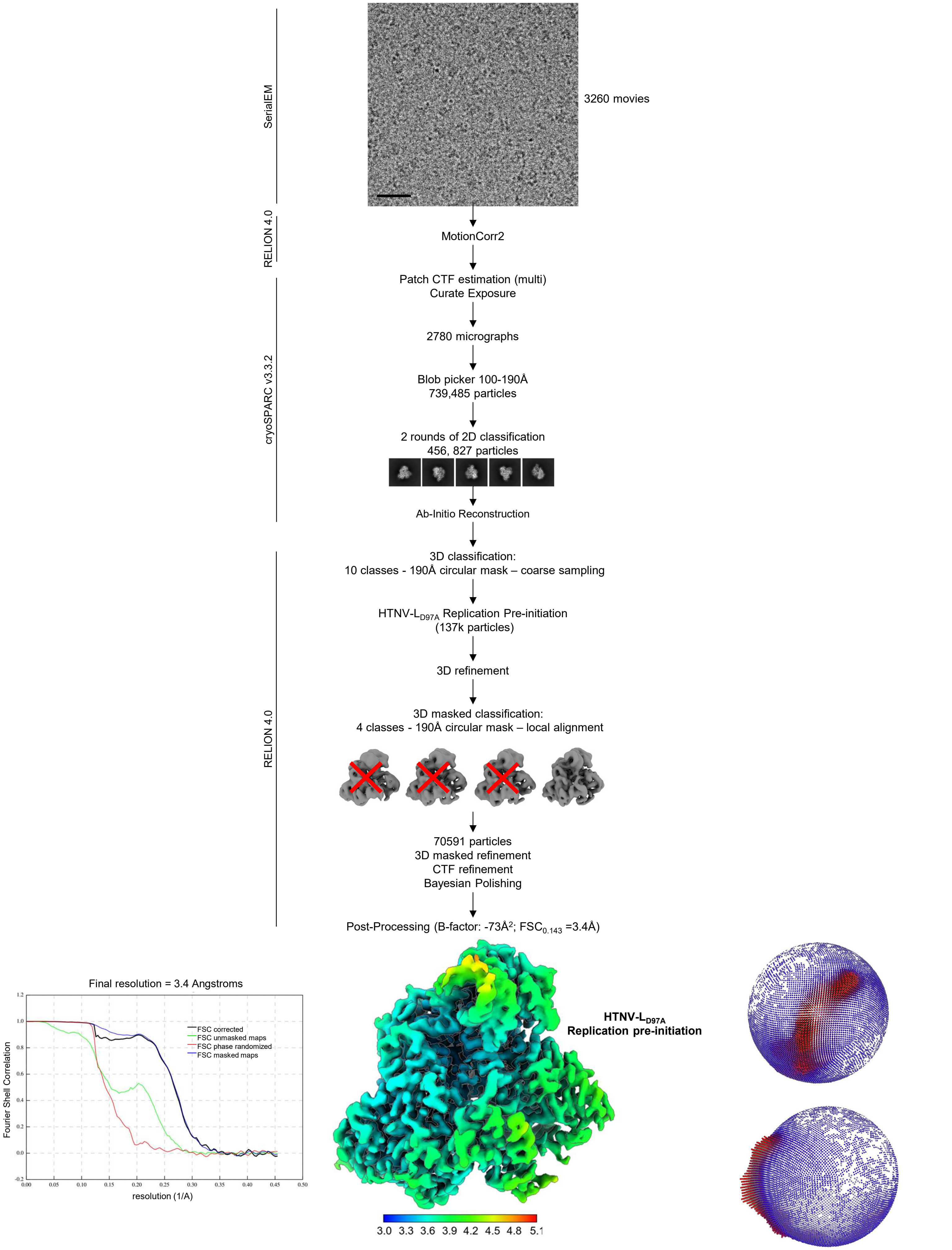

Supplementary Figure 9 Image processing strategy to obtain HTNV-L$_{D97A}$ cryo-EM map in pre-initiation state

Schematic representation of the image processing strategy. A representative image is displayed. The scale bar corresponds to 400 nm. 2D class averages, 3D class averages and the final reconstruction are displayed. Electron density maps are colored according to the local resolution. Fourier Shell Correlation curves (FSC) and angular distribution of particles used in the final reconstruction are displayed.

Supplementary Figure 10

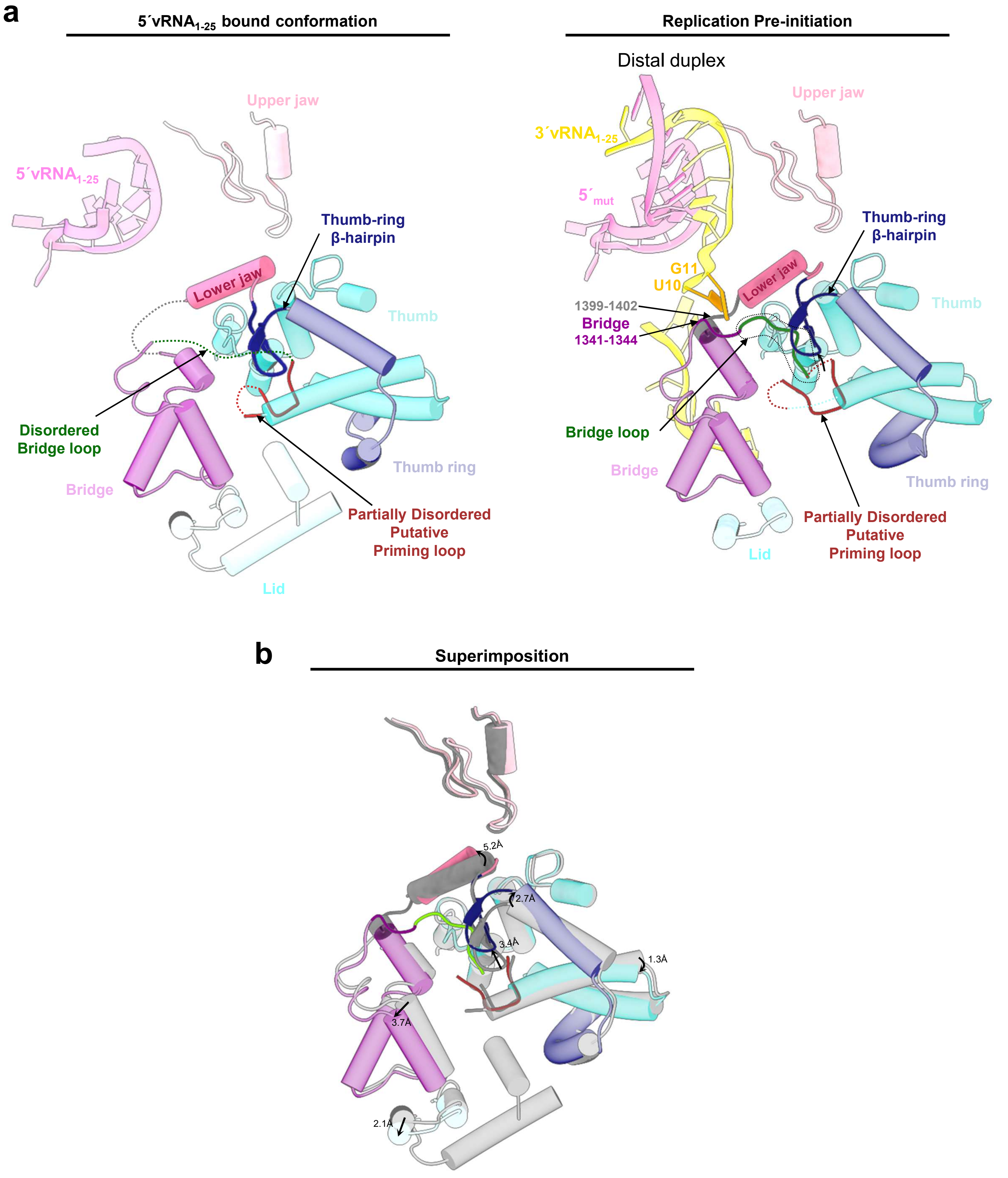

Supplementary Figure 10 Conformational changes between the 5'vRNA$_{1-25}$-bound map and the pre-initiation map

a Cut-away view cartoon representation of HTNV-L$_{D97A}$ regions that are changing in conformations in the 5'vRNA$_{1-25}$-bound and the pre-initiation maps. The RNA are shown as cartoon and labeled. The bridge loop, the thumb-ring β-hairpin, the residues 1399-1402 and 1341-1344 are respectively colored in green, dark blue, grey and purple. The other elements are colored as in **Fig. 1**. The bridge loop position is surrounded with a dotted line in the pre-initiation state. The movements between the 5'vRNA$_{1-25}$-bound and the pre-initiation conformations are indicated with small arrows on the pre-initiation panel. **b** Superimposition of the same regions with the 5'vRNA$_{1-25}$-bound colored in gray and the pre-initiation colored by domains. For clarity the RNA is not shown. Movements are indicated with arrows. Distances between the 5'vRNA$_{1-25}$-bound structure and the pre-initiation structure are indicated.

Supplementary Figure 11

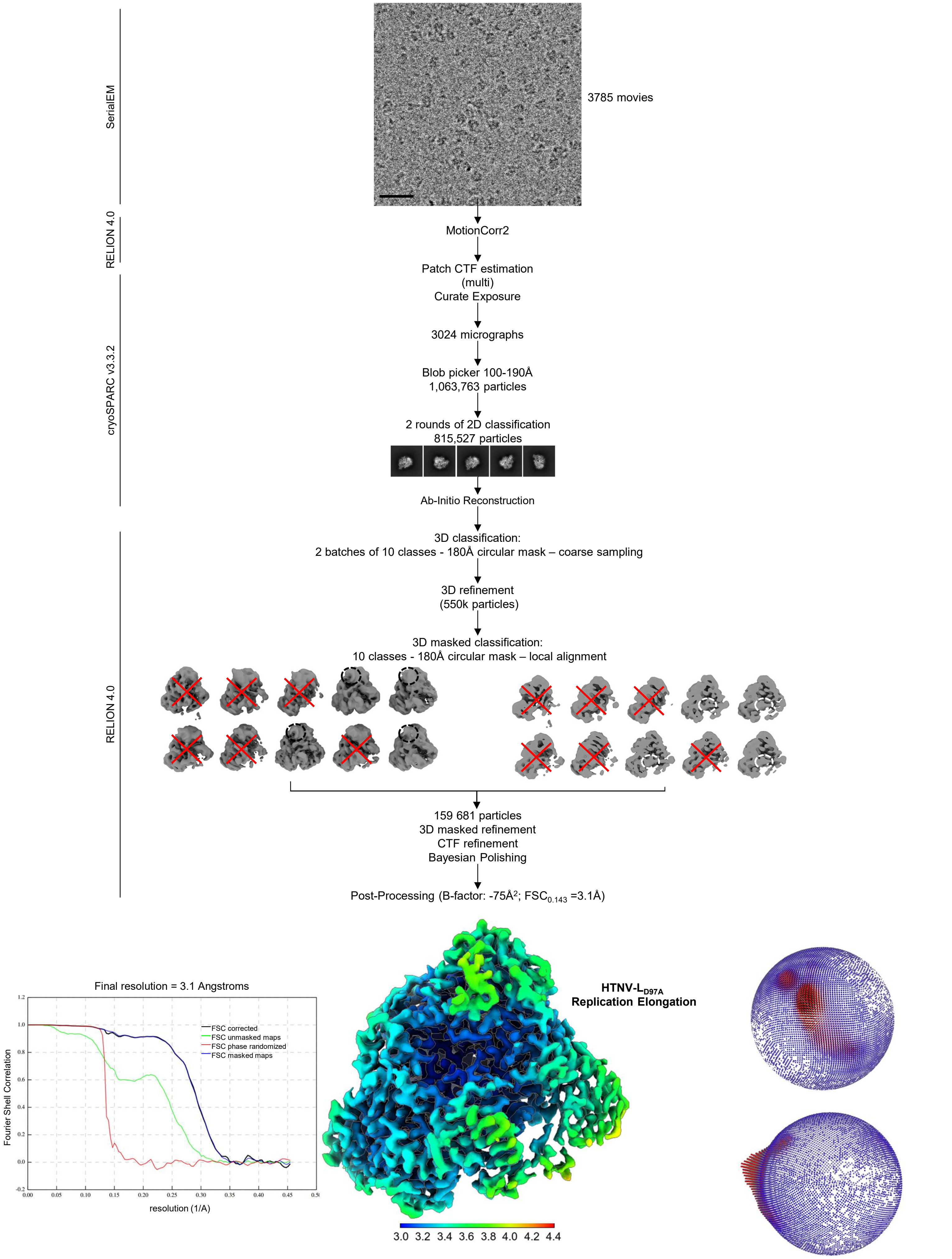

Supplementary Figure 11 Image processing strategy to obtain HTNV-L$_{D97A}$ cryo-EM map in elongation state

Schematic representation of the image processing strategy. A representative image is displayed. The scale bar corresponds to 400 nm. 2D class averages, 3D class averages and the final reconstruction are displayed. Electron density maps are colored according to the local resolution. Fourier Shell Correlation curves (FSC) and angular distribution of particles used in the final reconstruction are displayed.

Supplementary Figure 12

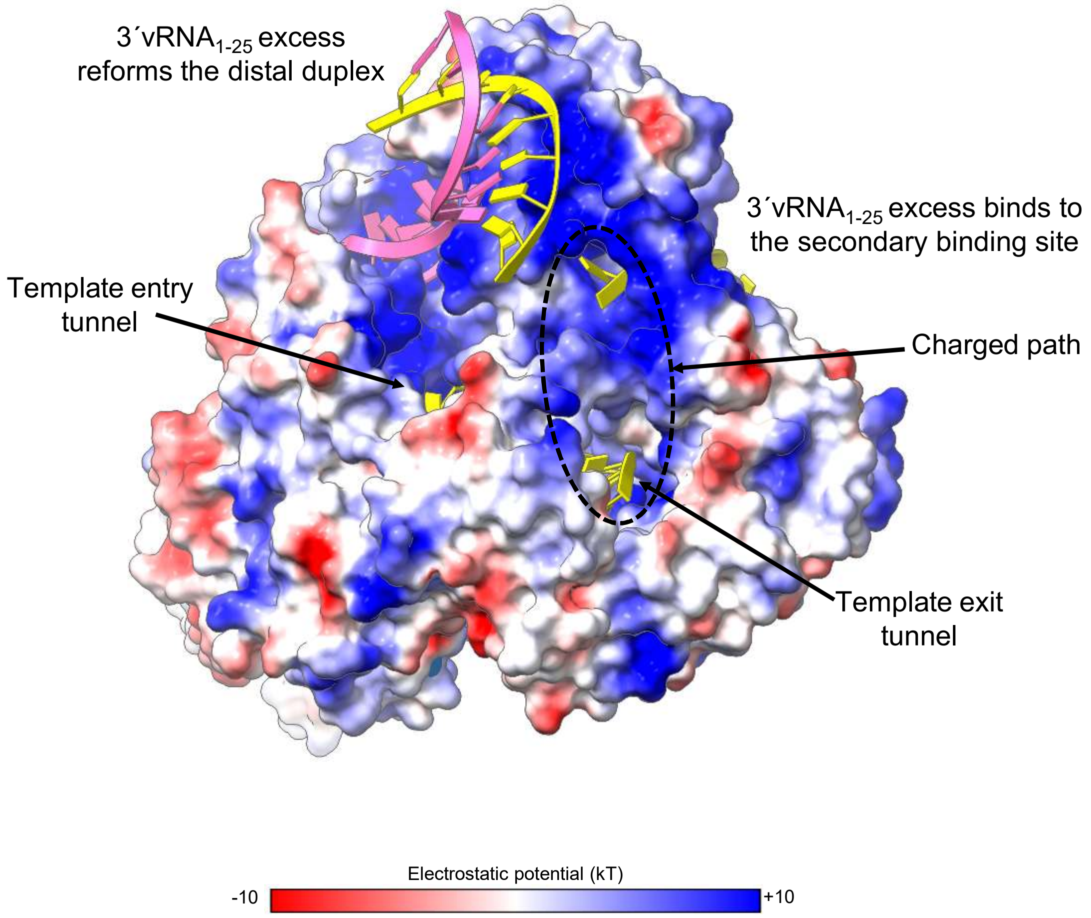

Supplementary Figure 12 Electrostatic surface of HTNV-L$_{D97A}$ in elongation state

Surface of HTNV-L$_{D97A}$ model in elongation state is colored according to its electrostatic potential. The 5'mut and 3'vRNA$_{1-25}$ are colored in pink and yellow respectively. The positively charged groove that connects the active site template exit tunnel to the 3'vRNA end secondary binding site is shown as a dotted line. The scale used to color the electrostatic surface is indicated at the bottom.

Supplementary Figure 13

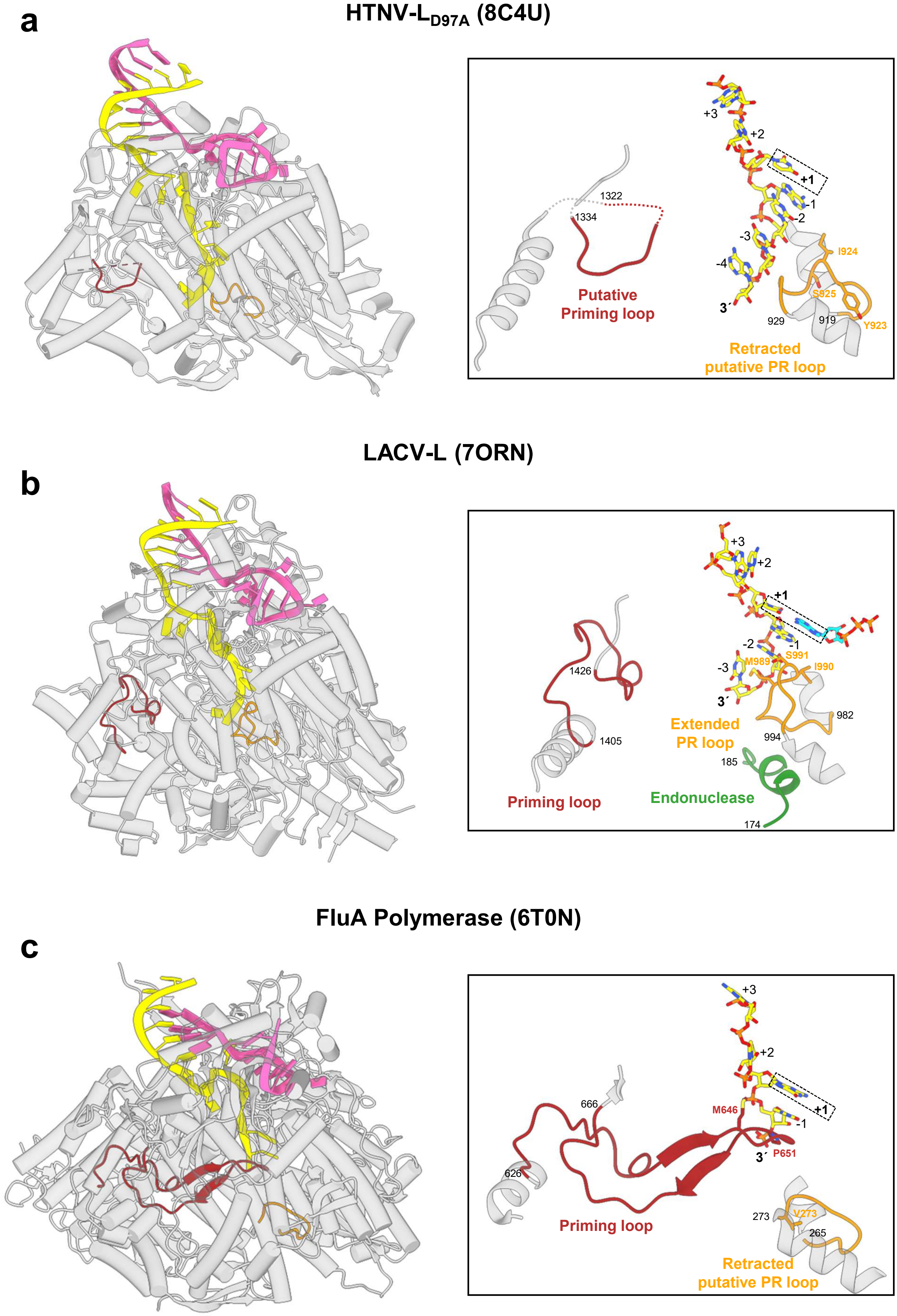

Supplementary Figure 13 Comparison of the putative priming and PR loops from different sNSV polymerases

Cryo-EM models of HTNV-L_{D97A} in pre-initiation (PDB 8C4U) (**a**), La Crosse virus polymerase (LACV-L) in initiation (PDB 7ORN) (**b**) and bat influenza polymerase in pre-initiation (PDB 6T0N) (**c**). On the left, cartoon representations of the polymerases colored in white except the putative prime-and-realign (PR) loop in orange and the putative priming loop in brown. The 5' and 3'vRNA are shown and respectively colored in pink and yellow. On the right, zoom on the putative PR loop, the putative priming loop and the 3'vRNA end. The 3'vRNA is shown as sticks and its position within the active site is labeled. Position +1 that corresponds to the position of the nucleotide to be incorporated is shown as a dotted line rectangle. The priming loop is located far from the 3'vRNA in HTNV-L_{D97A} and LACV-L. In HTNV-L_{D97A} the loop is partially disordered. In LACV-L the loop is ordered, located far from the active site, and is not involved in 3'vRNA positioning. In Influenza the priming loop protrudes up to the active site. Residues that interact with the 3'vRNA end are shown as sticks and labeled. Concerning the putative PR loop, it is present in HTNV-L_{D97A} and Influenza in a retracted conformation in the pre-initiation conformation. Important residues are shown as stick and labeled. In LACV-L the PR loop is in an elongated conformation in the initiation conformation. Residues that interact with the 3'vRNA end are shown as stick and labelled. The endonuclease helix that contributes to PR loop extension is shown in green.

Supplementary Figure 14

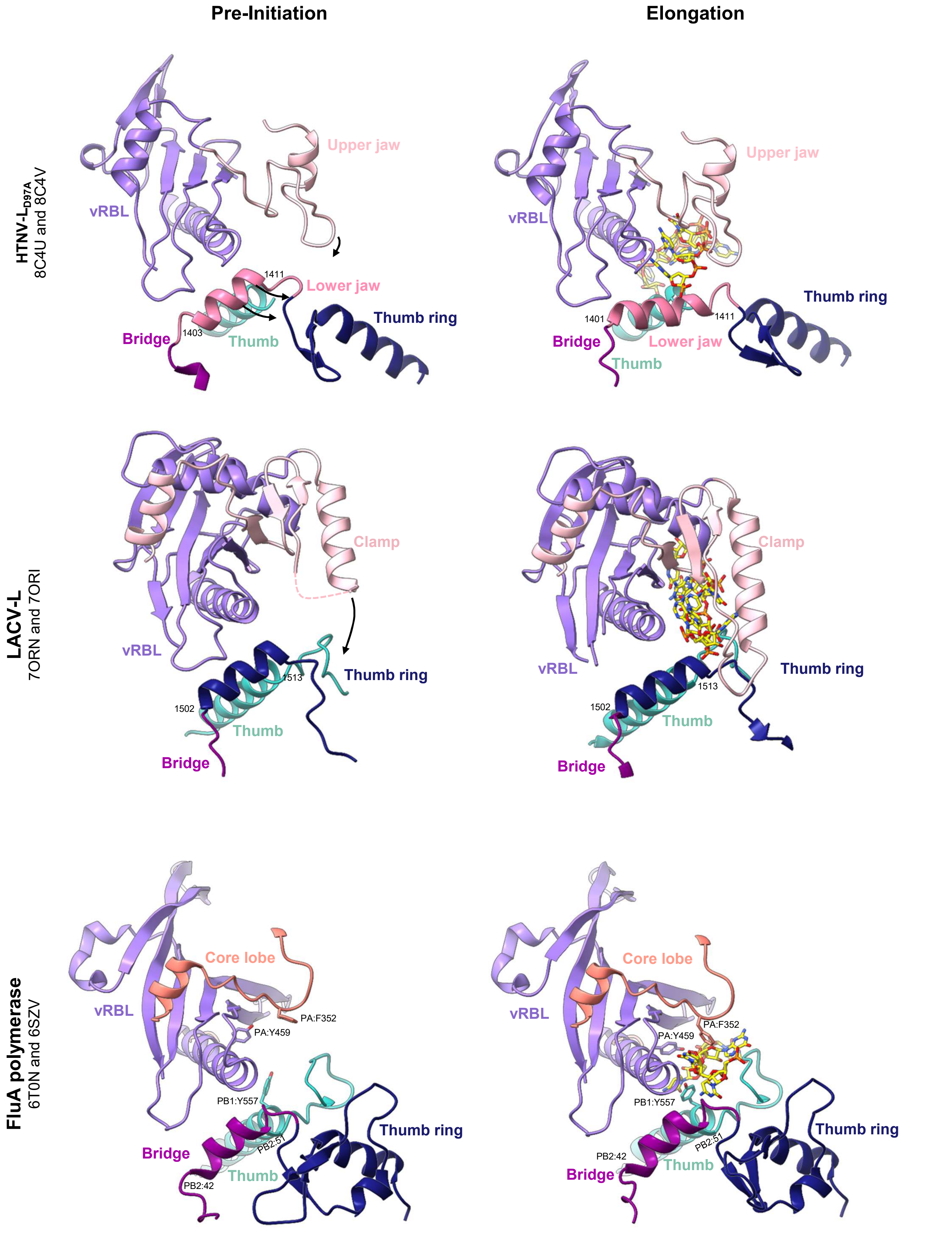

Supplementary Figure 14 Comparison of the 3′ secondary binding site from different segmented negative stranded RNA virus polymerases

3′ secondary binding site of HTNV-L, La Crosse virus polymerase (LACV-L) and bat influenza A polymerase in pre-initiation and elongation. Cartoon representation of the domains involved in 3′ secondary site formation colored as in **Fig.1** and labeled. The 3′vRNA present at elongation in the 3′ secondary binding site is shown as yellow stick. Main movements in HTNV-L$_{D97A}$and LACV-L between pre-initiation and elongation are shown with arrows. For bat influenza A, the side chains that change their positions to open the 3′end secondary binding site formation are labeled.

Supplementary Table 1 codon-optimized gene sequence of HTNV-L_{D97A} verified by DNA sequencing

ATGGGCCACCATCATCACCATCACGACTACGATATCCCCACGACAGAAAACTTGTACTTCCAGGGTATGGATAAGTACAGGGAAATTCATAACAAGCTAAA
GGAGTTTTCGCCCGGTACGCTGACTGCGGTCGAGTGTATCGATTACTTAGACCGTCTTTACGCCGTGCGCCACGACATAGTCGACCAAATGATTAAACACG
ACTGGTCTGACAATAAGGATAGTGAAGAGGCTATTGGGAAGGTACTGTTATTTGCCGGTGTGCCTTCTAACATAATCACCGCTCTGGAGAAGAAGATTATC
CCAAATCACCCAACTGGGAAGTCTCTGAAAGCATTTTTCAAAATGACCCCCGCTAACTACAAGATCTCTGGCACCACTATCGAATTTGTGGAAGTGACAGTG
ACTGCCGATGTTGACAAGGGCATCAGGGAGAAAAAGTTGAAGTACGAGGCCGGTTTAACGTATATCGAGCAGGAATTGCACAAGTTCTTTCTAAAGGGA
GAGATCCCCCAACCATATAAGATTACTTTCAACGTCGTGGCAGTCCGTACCGATGGATCCAATATCACTACCCAATGGCCTTCACGTCGGAACGATGGCGTA
GTCCAGTATATGAGGCTGGTCCAAGCCGAGATAAGCTACGTGAGAGAACATTTGATCAAGACGGAGGAGCGAGCGGCATTGGAGGCTATGTTCAACTTG
AAGTTCAACATCTCAACTCACAAGTCGCAACCATACTACATCCCCGATTACAAAGGAATGGAGCCGATCGGAGCAAACATTGAAGATTTGGTGGACTATAG
CAAAGACTGGTTAAGCCGCGCTCGCAACTTCTCGTTCTTCGAAGTCAAGGGAACGGCTGTGTTCGAATGCTTCAATAGTAACGAAGCAAATCACTGCCAGC
GCTATCCAATGTCGCGTAAGCCCCGTAACTTCCTACTCATTCAATGCAGTCTGATCACCTCATACAAGCCTGCAACTACTTTGTCTGACCAGATAGACAGTCG
TCGTGCTTGCTCATACATCCTCAATCTCATTCCTGACACTCCGGCCTCCTATCTTATCCACGACATGGCCTACCGTTACATTAATCTCACACGGGAGGACATG
ATTAACTATTACGCTCCGAGGATTCAGTTCAAGCAGACCCAGAACGTGCGTGAACCTGGCACCTTCAAATTGACTTCTAGTATGCTTAGGGCCGAGTCCAA
AGCAATGCTAGACTTACTGAATAATCATAAGTCTGGCGAGAAACACGGTGCCCAAATTGAATCCTTAAACATTGCTTCGCACATCGTACAATCCGAATCCGT
GTCACTCATTACGAAGATTTTGTCCGATCTAGAACTGAACATCACTGAACCTTCTACACAAGAGTATTCCACGACAAAGCACACCTACGTCGATACCGTTCTC
GACAAGTTTTTCCAAAATGAGACACAGAAGTATTTGATCGATGTGTTGAAAAAGACGACTGCATGGCACATCGGCCATCTGATACGCGATATCACGGAATC
ATTGATTGCCCATTCTGGATTGAAGAGAAGCAAGTACTGGTCACTCCATTCGTACAACAATGGAAACGTGATCTTATTCATCCTGCCCTCAAAATCTCTCGA
AGTAGCTGGCTCTTTTATCCGCTTTATCACCGTGTTCCGCATAGGTCCCGGCCTGGTAGACAAAGACAATCTCGACACTATTCTAATCGACGGTGACAGCCA
ATGGGGCGTGTCCAAAGTTATGTCGATCGACTTGAATCGTCTGCTTGCTTTGAATATCGCTTTTGAGAAAGCACTTATCGCAACCGCGACCTGGTTCCAGTA
CTACACAGAAGATCAGGGTCAGTTCCCACTGCAATACGCCATTCGCAGCGTGTTCGCAAATCATTTCCTCCTGGCTATCTGCCAAAAGATGAAACTGTGCGC
GATTTTCGATAACCTCCGATACCTCATCCCCGCAGTTACTTCTCTGTACTCCGGGTTCCCGTCACTGATCGAAAAGTTGTTCGAACGCCCTTTCAAGTCCTCTC
TGGAAGTGTACATCTACTACAACATCAAATCGCTCCTGGTGGCCCTCGCTCAAAACAACAAGGCTCGTTTTTACTCAAAGGTCAAGCTGCTGGGGCTGACC
GTAGACCAGTCGACGGTCGGAGCATCTGGCGTATACCCATCTTTCATGTCGAGGATTGTGTATAAGCACTACCGTAGTCTGATCTCCGAAGTTACGACGTG
CTTCTTCTTATTCGAGAAAGGTTTGCACGGCAACATGAACGAGGAAGCAAAGATACATTTGGAGACAGTGGAGTGGGCGCTCAAATTCCGAGAAAAGGAA
GAGAAGTACGGAGAGTCTCTTGTTGAAAATGGTTACATGATGTGGGAACTCAGAGCTAACGCCGAGTTGGCCGAACAGCAACTGTACTGCCAGGACGCTA
TCGAGCTGGCCGCAATCGAACTCAATAAGGTCTTGGCTACTAAGTCCTCGGTTGTCGCGAACAGCATCCTGTCCAAAAACTGGGAGGAGCCTTACTTCAGC
CAAACACGGAATATCTCTCTGAAGGGCATGTCAGGCCAGGTCCAAGAGGACGGACACTTAAGTTCCAGCGTAACTATTATCGAGGCTATTCGATACCTATC
GAACTCTAGGCATAACCCTAGCCTTTTGAAGCTCTACGAGGAAACGAGAGAGCAGAAGGCTATGGCCCGCATAGTACGCAAGTATCAGCGGACTGAAGCT
GACAGAGGGTTCTTTATCACAACTCTTCCCACACGCTGTAGACTCGAAATCATCGAGGACTACTATGACGCGATTGCGAAGAACATCAGCGAAGAGTATAT
CAGCTATGGAGGTGAAAAGAAGATCTTGGCAATTCAGGGAGCGTTGGAGAAGGCACTCAGATGGGCCAGCGGCGAATCTTTCATCGAGCTGAGCAACCA
TAAGTTCATCAGGATGAAGCGTAAACTCATGTACGTTAGTGCGGATGCTACAAAATGGAGCCCAGGCGACAACTCAGCTAAGTTCAGACGTTTCACCAGTA
TGCTGCATAACGGCCTCCCCAACAACAAACTGAAAAACTGTGTAATCGACGCGTTGAAACAGGTCTACAAGACCGACTTCTTTATGTCGCGCAAGCTGCGA
AACTACATCGACAGCATGGAGAGCTTAGACCCACACATAAAGCAATTCCTAGACTTCTTTCCCGACGGCCACCACGGCGAGGTCAAAGGTAATTGGCTGCA
GGGAAACCTAAACAAATGCTCCAGCCTTTTCGGAGTTGCAATGTCACTTTTGTTCAAGCAAGTGTGGACGAACCTCTTCCCAGAGTTGGACTGCTTTTTCGA
GTTCGCTCATCATTCAGACGACGCTCTCTTTATCTACGGATACCTAGAGCCGGTGGATGACGGAACAGATTGGTTCCTTTTTGTTTCACAACAGATTCAGGC
CGGTCACCTACACTGGTTTTCCGTGAACACTGAGATGTGGAAGTCCATGTTTAACTTACACGAGCATATCCTGCTCCTAGGGTCCATTAAGATTAGCCCTAA
GAAAACAACCGTAAGCCCGACGAACGCTGAGTTCCTCAGCACTTTCTTCGAGGGATGCGCAGTTTCTATACCTTTCGTTAAGATTCTCCTCGGAAGCCTGAG
TGACCTCCCCGGTTTAGGCTACTTTGATGATCTGGCTGCTGCGCAATCACGTTGTGTGAAGGCTCTCGACCTTGGTGCTTCCCCGCAAGTCGCGCAACTGGC
CGTGGCATTGTGTACATCGAAGGTAGAAAGGTTATACGGTACAGCTCCGGGAATGGTCAACCACCCCGCTGCCTATCTTCAAGTGAAGCACACTGATACAC
CAATCCCACTGGGTGGAAACGGCGCTATGTCGATAATGGAACTGGCGACCGCCGGAATCGGAATGTCTGACAAAAATCTGTTAAAGCGAGCATTGTTAGG
ATACTCACACAAGAGGCAGAAAAGTATGCTCTACATTTTGGGCCTGTTCAAGTTCCTCATGAAGCTAAGCGATGAAACCTTCCAGCACGAACGTCTGGGAC
AGTTTTCGTTCATCGGTAAAGTCCAATGGAAAATCTTTACCCCGAAGAGTGAGTTTGAGTTCGCCGACATGTACACCTCCAAGTTTCTGGAACTGTGGTCAT
CTCAGCATGTTACCTATGACTACATCATCCCAAAGGGGAGGGACAACCTCCTCATCTACCTCGTACGCAAGCTGAACGATCCTTCGATCGTGACTGCTATGA
CCATGCAGAGCCCCTTGCAGCTTAGGTTTAGGATGCAGGCCAAGCAGCACATGAAGGTTTGCCGCTTAGACGGAGAATGGGTGACGTTTAGGGAGGTCCT
CGCGGCTGCCAACTCTTTTGCCGAGAACTACAGTGCCACAAGCCAGGATATGGACCTTTTTCAGACGCTGACCTCGTGCACCTTTTCTAAGGAATACGCATG
GAAAGATTTCCTCAACGGTATTCACTGCGACGTCATTCCGACAAAACAGGTCCAGCGCGCAAAGGTAGCTCGAACGTTCACGGTGCGCGAAAAGGACCAG
ATTATCCAGAATAGTATCCCGGCTGTCATCGGTTACAAATTTGCGGTGACAGTAGAGGAGATGTCCGACGTGCTCGATACAGCGAAGTTTCCCGACTCTCT
CTCGGTGGATCTAAAGACTATGAAAGACGGAGTATACCGCGAATTGGGTTTAGACATCTCACTGCCCGATGTCATGAAACGTATCGCACCCATGTTGTACA
AAAGTTCCAAGTCTAGGGTCGTGATTGTCCAAGGAAACGTAGAGGGTACGGCCGAAGCAATTTGTCGCTACTGGCTCAAATCGATGTCACTTGTTAAAACG
ATTCGTGTCAAACCACACAAGGAGGTCTTGCAAGCAGTCTCGATTTTCAACCGCAAAGAGGACATTGGTCAACAGAAGGACCTTGCGGCCCTTAAGTTGTG
CATCGAAGTGTGGCGTTGGTGTAAGGCTAACTCGGCTCCCTACAGAGACTGGTTCCAAGCTCTCTGGTTCGAGGATAAGACCTTCTCTGAGTGGCTCGACA
GGTTCTGCCGTGTGGGGGTTCCTCCAATCGATCCGGAAATCCAATGTGCTGCCCTCATGATCGCGGACATCAAAGGAGATTACAGTGTGTTGCAACTGCAG
GCGAACAGGAGAGCCTACAGCGGTAAACAATATGACGCGTACTGTGTCCAGACATATAACGAGGTGACGAAACTGTATGAGGGCGATCTAAGGGTGACC
TTCAATTTTGGGCTTGATTGTGCCAGGCTTGAGATATTCTGGGACAAAAAGGCATACATTCTCGAAACCTCTATCACCCAGAAACACGTGTTGAAGATTATG
ATGGACGAGGTTTCCAAGGAGCTTATCAAGTGCGGCATGCGCTTCAATACCGAACAGGTGCAAGGTGTCCGTCACATGGTTCTGTTCAAAACAGAGAGTG
GTTTCGAATGGGGAAAGCCTAACATCCCCTGCATCGTGTATAAGAACTGTGTGTTGCGTACCTCGTTACGCACAACACAGGCCATTAACCACAAGTTCATG
ATCACCATAAAGGACGATGGCCTCCGTGCCATTGCACAACACGATGAGGACAGCCCTAGATTCCTTCTCGCCCACGCCTTCCATACCATACGTGATATCCGC
TATCAGGCCGTGGACGCAGTGTCGAATGTCTGGTTCATCCACAAAGGGGTCAAACTGTACTTGAACCCAATTATCAGTAGCGGTCTGCTGGAGAACTTTAT
GAAAAACCTCCCCGCTGCGATTCCACCAGCGGCCTACAGCCTCATCATGAACCGTGCTAAGATCTCAGTAGATCTCTTCATGTTCAACGATCTGCTTAAGCT
AATCAATCCTCGCAACACCCTCGATCTGTCTGGTCTTGAAACGACTGGCGACGAGTTTTCGACAGTTTCCTCTATGAGTAGTCGTTTGTGGTCTGAAGAGAT
GTCCTTAGTCGACGACGACGAGGAGCTGGATGATGAGTTCACCATTGATTTGCAAGACGTAGATTTCGAGAACATCGATATTGAGGCCGATATCGAACACT
TTTTACAAGACGAATCCTCCTACACCGGTGATCTACTTATCTCGACTGAGGAAACCGAGAGCAAAAAGATGCGCGGAATTGTGAAAATTCTGGAGCCGGTC
AGACTGATAAAGAGCTGGGTATCACGAGGCCTGAGTATCGAGAAAGTGTACAGTCCAGTGAATATCATATTGATGTCTCGATACATCTCAAAGACGTTTAA
CTTGTCAACTAAGCAAGTTTCACTGCTGGACCCTTACGACTTGACAGAACTCGAATCAATCGTCCGCGGCTGGGGTGAATGTGTTATTGACCAATTCGAATC
CTTAGATAGGGAAGCTCAGAATATGGTGGTGAACAAAGGGATTTGCCCAGAAGATGTGATCCCCGACTCGCTGTTCTCCTTCCGTCACACCATGGTCCTGC
TAAGACGACTTTTCCCGCAAGACTCAATCAGTAGCTTCTACTAATAA

Supplementary Table 2. Cryo-EM data collection, refinement and validation statistics of HTNV-L$_{D97A}$ structures

	HTNV-L$_{D97A}$ apo<br><br>PDB 8C4S EMD-16427	HTNV-L$_{D97A}$ 5'vRNA$_{1-25}$-bound<br><br>PDB 8C4T EMD-16428	HTNV-L$_{D97A}$ pre-initiation<br><br>PDB 8C4U, EMD-16429	HTNV-L$_{D97A}$ elongation<br><br>PDB 8C4V, EMD-16430
**Data collection and processing**				
Microscope	Thermo Fisher Scientific Glacios	Thermo Fisher Scientific Glacios	Thermo Fisher Scientific Glacios	Thermo Fisher Scientific Glacios
Camera	Gatan K2 Summit	Gatan K2 Summit	Gatan K2 Summit	Gatan K2 Summit
Magnification	36000	36000	36000	36000
Voltage (kV)	200	200	200	200
Number of frames	50	50	50	50
Electron exposure (e^-/A^2)	50	50	50	50
Defocus range (μm)	-0.8 to -2.2	-0.8 to -2.2	-0.8 to -2.2	-0.8 to -2.2
Pixel size (Å)	1.145	1.145	1.1	1.1
Symmetry imposed	C1	C1	C1	C1
Initial/Final micrographs (no.)	2856/2154	2547/2112	3260/2780	3785/3024
Final particles (no.)	105 717	174 977	70 591	159 681
Map resolution (Å) 0.143	3.3	3.2	3.3	3.1
FSC threshold	0.143	0.143	0.143	0.143
Map resolution range (Å)	3.1 - 4.4	3.0-6.9	3.3-6.1	3.0-8.5
**Refinement**				
Model resolution (Å) 0.5 FSC threshold	3.2	3.3	3.4	3.1
Map sharpening B factor ($Å^2$)	-75	-82	-75	-75
Model composition				
Protein residues	1277	1268	1260	1309
Nucleotide residues	0	12	38	56
Ligands	0	1	1	1
Water	0	0	0	0
B-factor ($Å^2$, min-max (mean))				
Protein	6.33-137.15 (65.75)	8.72-111.33 (50.53)	11.74-146.73 (68.69)	9.18-105.39 (34.70)
Nucleotides	0	36.06-112.23 (70.65)	33.03-166.60 (99.12)	4.36-166.9- (60.66)
Ligands	0	41.28-41.28 (41.28)	43.63-43.63 (43.63)	14.82-14.82 (14.82)
R.m.s deviations				
Bond lengths (Å)	0.004	0.003	0.004	0.004
Bond angles (°)	0.550	0.678	0.716	0.572
Validation				
MolProbity score	1.57	1.62	1.70	1.95
Clashscore	8	8	7	13
Poor rotamers (%)	0.18	0.18	0.27	0.26
Ramachandran plot				
Favored (%)	97.28	97.10	96.46	95.91
Allowed (%)	2.72	2.90	3.46	4.02
Disallowed (%)	0	0	0	0

Supplementary Table 3 Protein-RNA interaction in the 3'vRNA end secondary binding site

		Hydrophobic	H-bond	Stacking	Pocket
A1	base	I464, K468, W473	Q460		Pocket 1
	ribose	S1234, A1230			
	phosphate		H477, R480		
U2	base	P304, A305	R480	A4 base	Pocket 2
	ribose		H477		
	phosphate				
C3	base	P1244, G1245			Towards protein exterior
	ribose				
	phosphate		U2 base		
A4	base	M1246, P304	R1238, U5 ribose	U2 base	Pocket 2
	ribose	G1245			
	phosphate	T306, T307			
U5	base	A305, T307, Y353		C6 base	Pocket 3
	ribose	A305	U2 base, A4 base, C6 ribose		
	phosphate		H1413		
C6	base		A488	U5 base	Pocket 3
	ribose		Q1412, U5 ribose, A7 ribose		
	phosphate		R494, R356		
A7	base	A488, G491	R494, C6 ribose	F1406, R494	Pocket 4
	ribose	F1406	Q1409		
	phosphate				
