# Supplementary material for: Structures of active Hantaan virus polymerase uncover the mechanisms of Hantaviridae genome replication
Source: Nat Commun. 2023 May 23;14:2954. doi: 10.1038/s41467-023-38555-w (PMC10206067; doi:10.1038/s41467-023-38555-w)
Supplement: Supplementary file 4 — Description of Additional Supplementary Files [file 41467_2023_38555_MOESM4_ESM.pdf]

Description of Additional Supplementary Files:

Supplementary Data 1: Multiple alignment of Hantaviridae polymerases Polymerase sequences of Hantaan virus (HTNV-L), Sin Nombre virus (SINV-L), Andes virus (ANDV-L), Puumala virus (PUUM-L) and Tula virus (TULV-L) were aligned. The domain names and the active site motifs are displayed and colored on top of the multiple alignment.

Supplementary Data 2: List of all reagent/resource with their source/supplier and identifier.

Supplementary Movie 1: Conformational changes between HTNV-L apo and 5'vRNA-bound structures.
