# Supplementary material for: Structures of active Hantaan virus polymerase uncover the mechanisms of Hantaviridae genome replication
Source: Nat Commun. 2023 May 23;14:2954. doi: 10.1038/s41467-023-38555-w (PMC10206067; doi:10.1038/s41467-023-38555-w)

Supplementary Data (1/3)

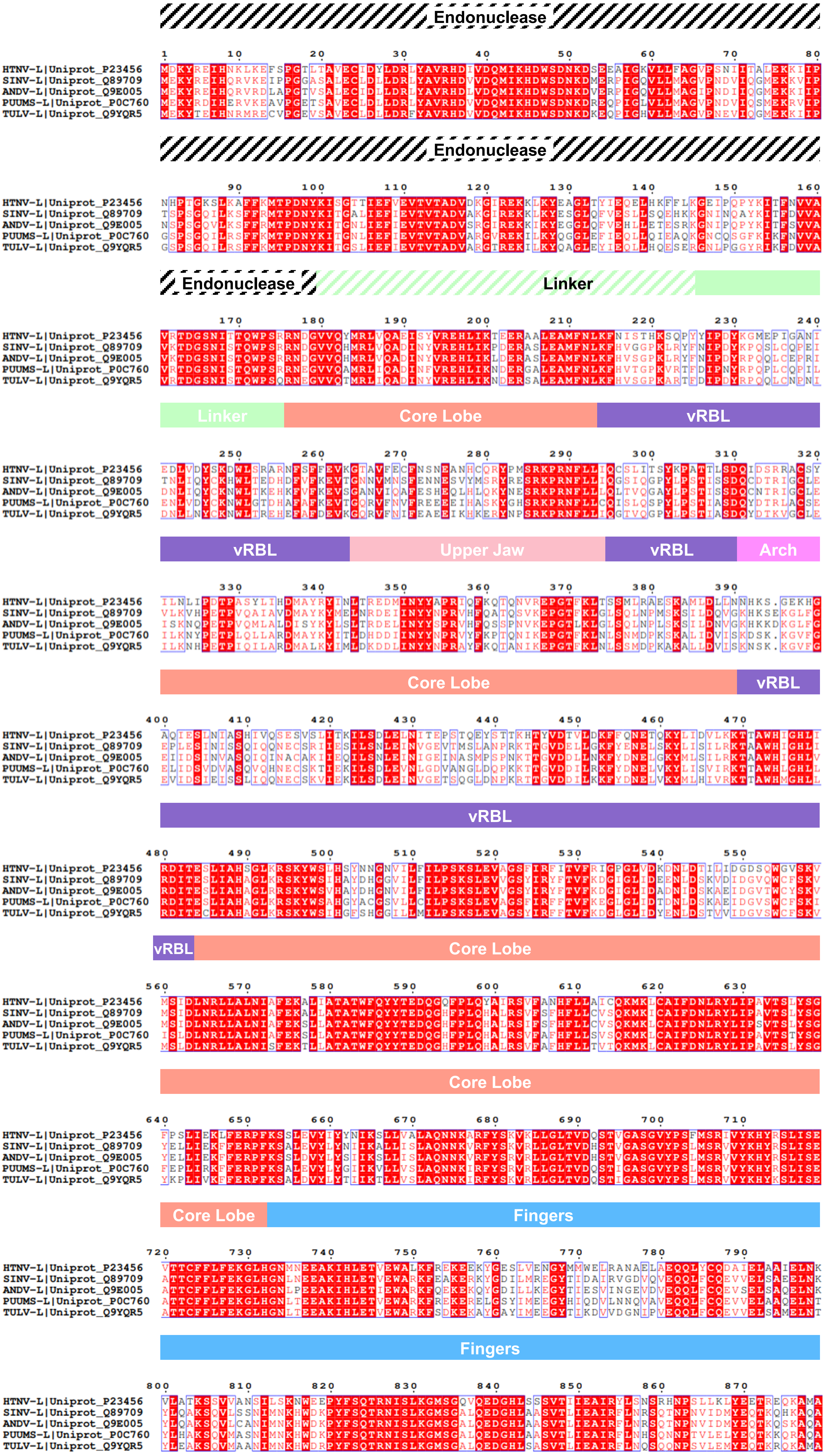

Supplementary Data (2/3)

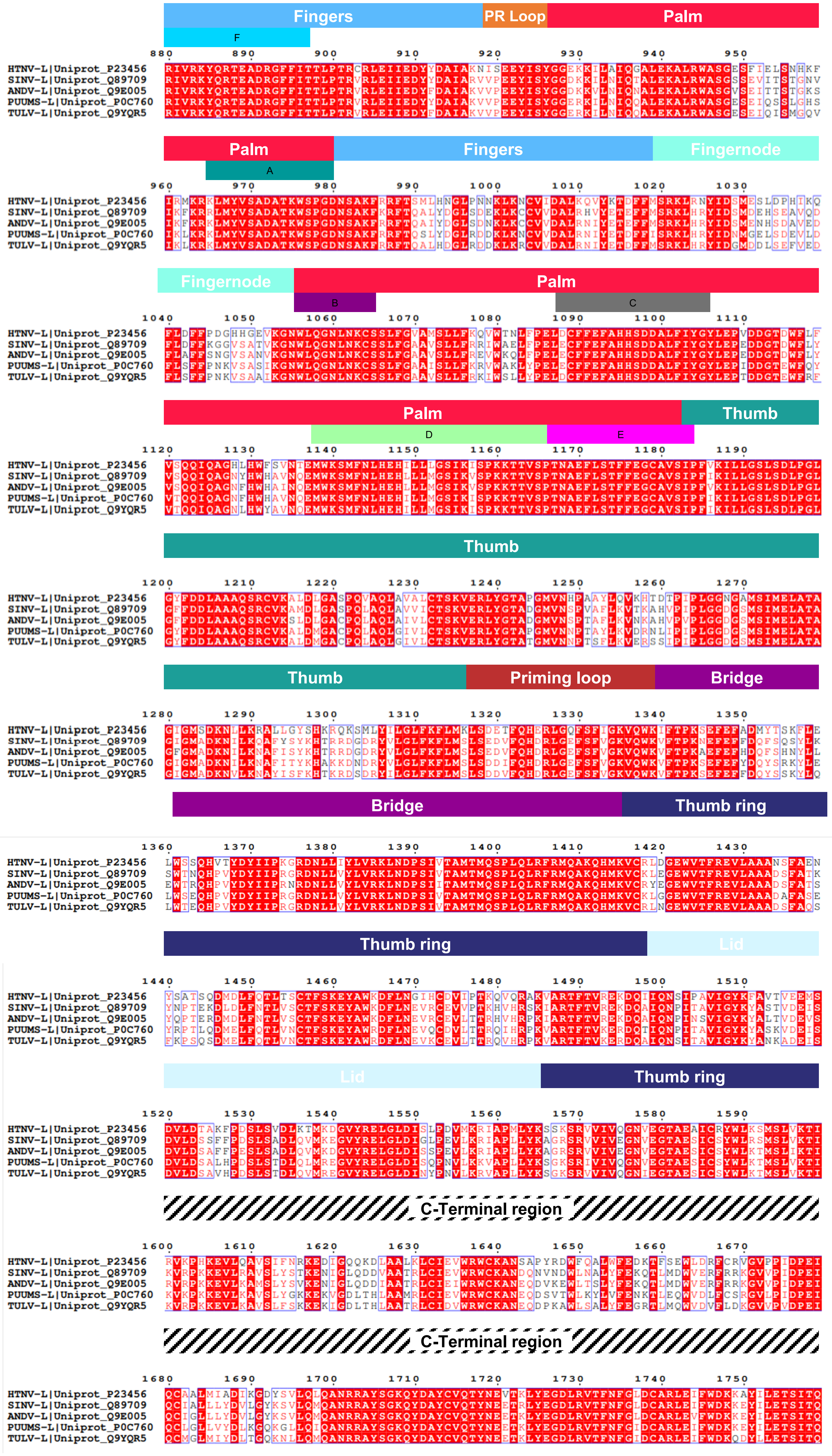

Supplementary Data (3/3)

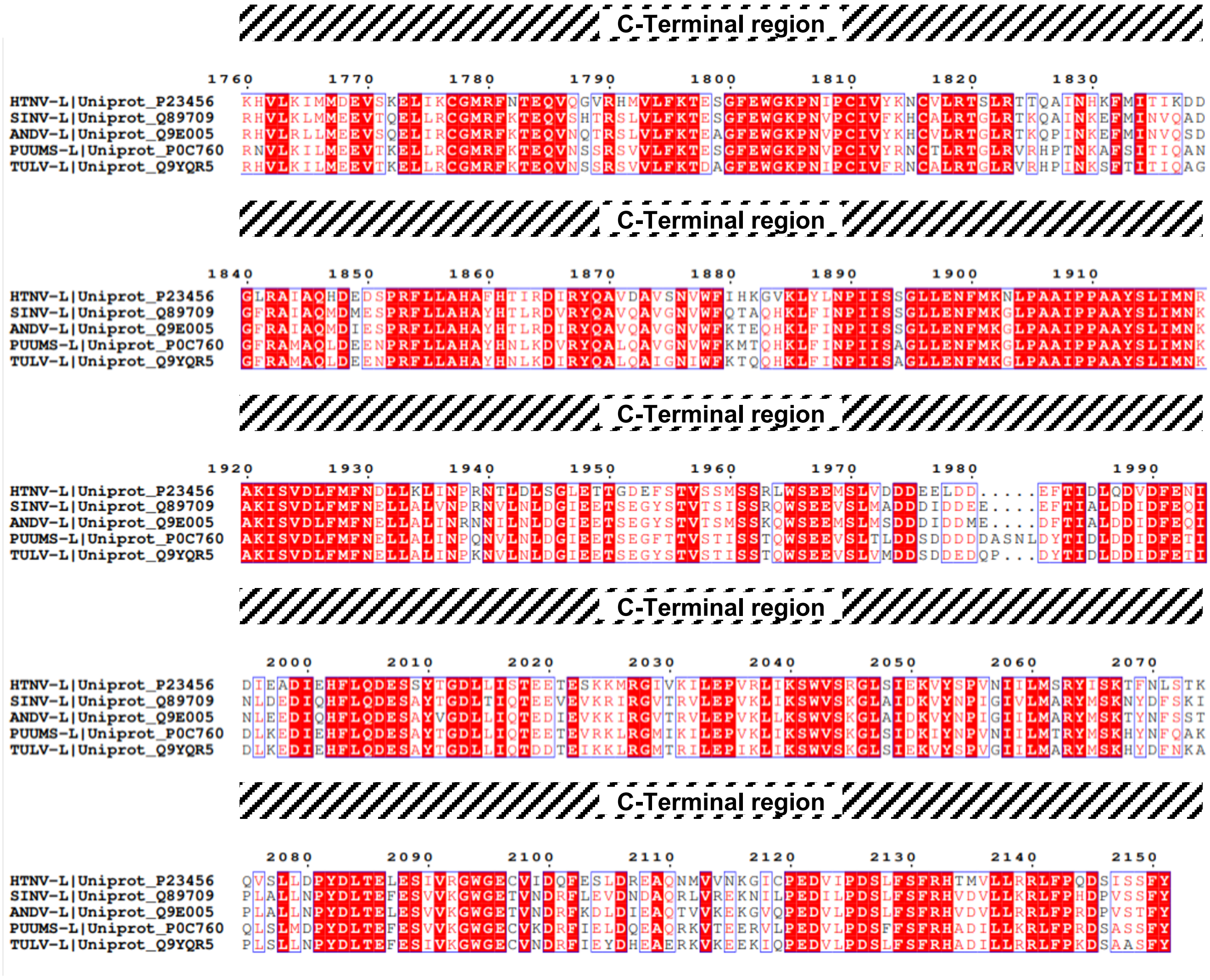

Supplement: Supplementary file 5 — Supplementary Dataset 1 [file 41467_2023_38555_MOESM5_ESM.pdf]
